# Broad AOX expression in a genetically tractable mouse model does not disturb normal physiology

**DOI:** 10.1242/dmm.027839

**Published:** 2017-02-01

**Authors:** Marten Szibor, Praveen K. Dhandapani, Eric Dufour, Kira M. Holmström, Yuan Zhuang, Isabelle Salwig, Ilka Wittig, Juliana Heidler, Zemfira Gizatullina, Timur Gainutdinov, German Mouse Clinic Consortium, Helmut Fuchs, Valérie Gailus-Durner, Martin Hrabě de Angelis, Jatin Nandania, Vidya Velagapudi, Astrid Wietelmann, Pierre Rustin, Frank N. Gellerich, Howard T. Jacobs, Thomas Braun

**Affiliations:** 1 Institute of Biotechnology, FI-00014 University of Helsinki, Finland; 2 BioMediTech and Tampere University Hospital, FI-33014 University of Tampere, Finland; 3 Max Planck Institute for Heart and Lung Research, Cardiac Development and Remodelling (Department I), Bad Nauheim D-61231, Germany; 4 Functional Proteomics, SFB 815 Core Unit, Faculty of Medicine, Goethe-University, Frankfurt am Main D-60590, Germany; 5 German Center of Cardiovascular Research (DZHK), Partner site RheinMain, Frankfurt, Germany; 6 Cluster of Excellence “Macromolecular Complexes”, Goethe-University, Frankfurt am Main D-60590, Germany; 7 Leibniz Institute for Neurobiology, Magdeburg D-39118, Germany; 8 German Mouse Clinic, Institute of Experimental Genetics, Helmholtz Zentrum München, German Research Center for Environmental Health GmbH, Ingolstaedter Landstrasse 1, Neuherberg 85764, Germany; 9 Chair of Experimental Genetics, Center of Life and Food Sciences Weihenstephan, TU Munich, Emil-Erlenmeyer-Forum 2, Freising-Weihenstephan 85350, Germany; 10 Member of German Center for Diabetes Research (DZD), Ingolstaedter Landstrasse 1, Neuherberg 85764, Germany; 11 Institute for Molecular Medicine Finland, FI-00014 University of Helsinki, Finland; 12 INSERM UMR 1141 and Université Paris 7, Hôpital Robert Debré, Paris 75019, France; 13 Department of Neurology, Otto-von-Guericke-University, Magdeburg D-39120, Germany

**Keywords:** Mitochondria, Mitochondrial disease, Respiratory chain, Alternative oxidase

## Abstract

Plants and many lower organisms, but not mammals, express alternative oxidases (AOXs) that branch the mitochondrial respiratory chain, transferring electrons directly from ubiquinol to oxygen without proton pumping. Thus, they maintain electron flow under conditions when the classical respiratory chain is impaired, limiting excess production of oxygen radicals and supporting redox and metabolic homeostasis. AOX from *Ciona intestinalis* has been used to study and mitigate mitochondrial impairments in mammalian cell lines, *Drosophila* disease models and, most recently, in the mouse, where multiple lentivector-AOX transgenes conferred substantial expression in specific tissues. Here, we describe a genetically tractable mouse model in which *Ciona* AOX has been targeted to the *Rosa26* locus for ubiquitous expression. The *AOX^Rosa26^* mouse exhibited only subtle phenotypic effects on respiratory complex formation, oxygen consumption or the global metabolome, and showed an essentially normal physiology. AOX conferred robust resistance to inhibitors of the respiratory chain *in organello*; moreover, animals exposed to a systemically applied LD50 dose of cyanide did not succumb. The *AOX^Rosa26^* mouse is a useful tool to investigate respiratory control mechanisms and to decipher mitochondrial disease aetiology *in vivo*.

## Abstract (precis)

**Summary:** Previous limitations are overcome in this first genetically tractable mouse model expressing invertebrate alternative oxidase, AOX, which can suppress pathological stresses in the mitochondrial respiratory chain.

## INTRODUCTION

**Fig. 1.**
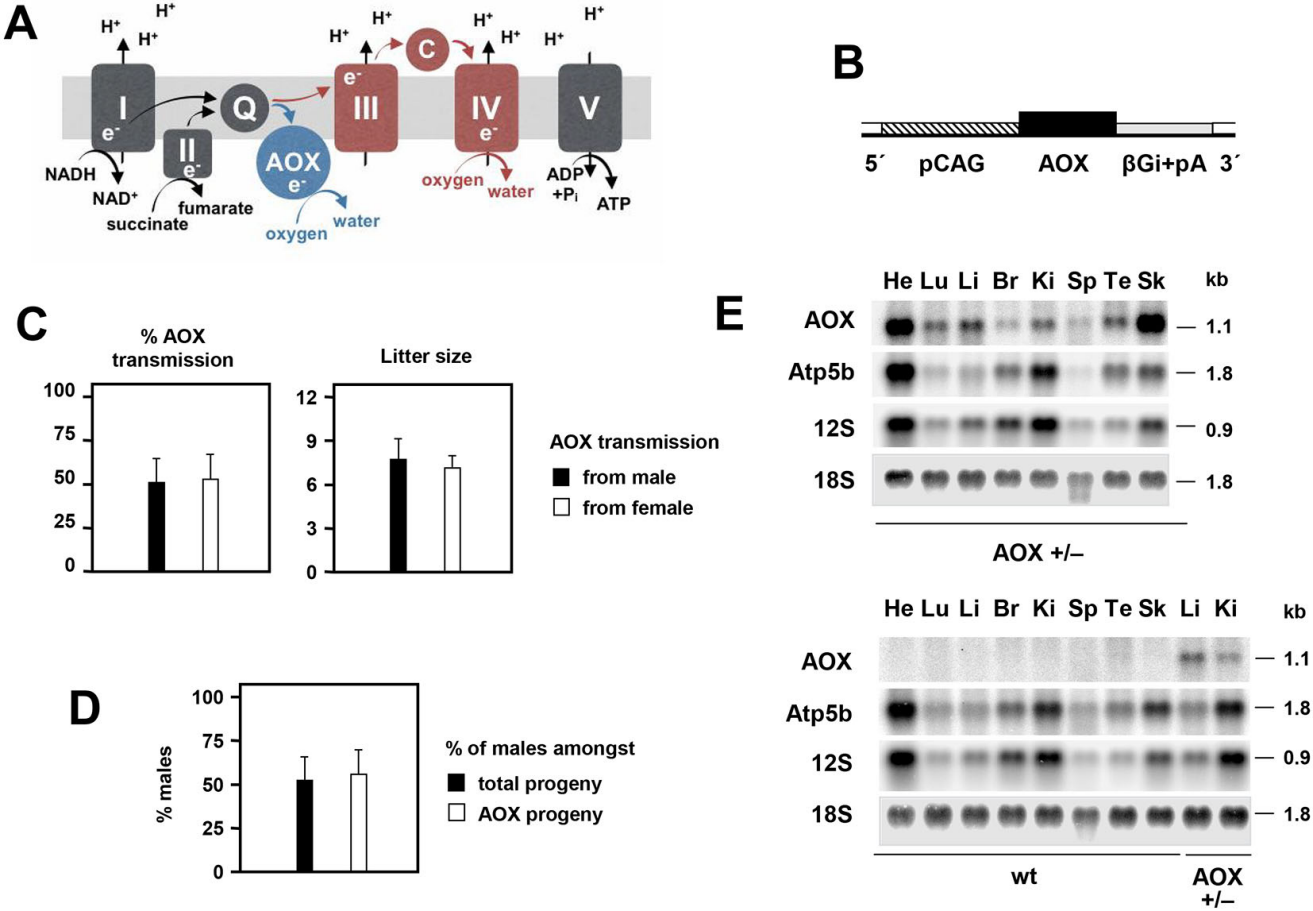
**Construction and characterization of *AOX^Rosa26^* mice.** (A) Schematic diagram of the mitochondrial OXPHOS system, showing the five standard OXPHOS complexes (I-V), the diffusible electron carriers ubiquinone (Q) and cytochrome c (c), and the passage of electrons and protons resulting ultimately in the synthesis of ATP from ADP and inorganic phosphate (Pi). The additional presence of AOX, whether supplied transgenically or in organisms naturally endowed with it, provides an alternative route for the reoxidation of ubiquinol by molecular oxygen, without proton pumping. (B) Schematic diagram of inserted Rosa26-AOX expression construct, following removal of additional elements (i.e. DTA negative selectable marker upon targeted integration, and neomycin resistance cassette following FRT-mediated excision *in vivo*). Remaining elements are the CAG promoter, AOX coding sequence and β-globin intron and poly(A) addition signal (βGi+pA). For full details see Fig. S1A. (C) Transmission rate of *AOX* transgenes (based on PCR) and litter sizes, according to sex of *AOX*-hemizygous parent. Transmission rates from male (*n*=93, 12 crosses) and from female (*n*=43, 6 crosses) were not significantly different from each other (Student's *t*-test, *P*>0.05, mean±s.d.) or from Mendelian expectation of 50% (chi-squared test). Litter sizes produced by *AOX*-hemizygous males and females also showed no significant difference (Student's *t*-test, *P*>0.05). (D) Sex (% of males) of transgenic and wild-type progeny of hemizygous *AOX^Rosa26^* mice (*n*=136, 18 crosses), again showing no significant differences (Student's *t*-test, *P*>0.05, mean±s.d.). (E) Northern blot showing *AOX* expression in RNA (10 μg) from tissues of one-year-old, male, hemizygous *AOX^Rosa26^* mice and wild-type (wt) littermate controls: He, heart; Lu, lung; Li, liver; Br, brain; Ki, kidney; Sp, spleen; Te, testis; Sk, skeletal muscle. The blot was reprobed for *Atp5b* mRNA as well as mitochondrial 12S and cytosolic 18S rRNAs as loading controls. RNA molecular weights were extrapolated from rRNA migration in the ethidium bromide-stained gel.

The mitochondrial system for oxidative phosphorylation (OXPHOS) comprises four multisubunit complexes supporting stepwise respiratory electron flow from primary electron acceptors to oxygen, and a fifth complex (ATP synthase) that uses the proton gradient thereby generated across the inner mitochondrial membrane to synthesize ATP. In many lower organism and plants, alternative oxidases (AOXs) are expressed that branch the mitochondrial respiratory chain, thus transferring electrons directly from ubiquinol to oxygen in a non-proton-motive manner. AOXs are absent in mammals (Young et al., 2013) (Fig. 1A). Their main physiological role is to maintain electron flow under conditions when the classical respiratory chain is impaired, limiting excess production of oxygen radicals and supporting redox and metabolic homeostasis. Because AOX is also found in some invertebrate phyla (McDonald et al., 2009), we have proposed that its expression in commonly studied animal models could be used to elucidate the pathophysiology underlying mitochondrial OXPHOS disorders, providing a rational basis for its eventual implementation in therapeutic applications (Rustin and Jacobs, 2009; El-Khoury et al., 2014).

In earlier studies, AOX from the tunicate *Ciona intestinalis*, a sister group to the vertebrates, was shown to be expressible and catalytically active in human cells (Hakkaart et al., 2006). It was found to alleviate the deleterious consequences of toxic or pathological inhibition of the downstream portion of the mitochondrial respiratory chain (Hakkaart et al., 2006; Dassa et al., 2009), specifically OXPHOS complexes III (cIII) and IV (cIV), which AOX bypasses. A cDNA encoding *Ciona* AOX was subsequently shown to be ubiquitously expressible in *Drosophila*, without eliciting any harmful phenotypic effects (Fernandez-Ayala et al., 2009). In the fly, AOX expression was able to compensate a range of pathological phenotypes at the whole-organism level, including lethality caused by OXPHOS poisons such as antimycin A or cyanide (Fernandez-Ayala et al., 2009), locomotor disturbance or neurodegeneration caused by cIV knockdown (Kemppainen et al., 2014) or other causes of neurodegeneration mimicking Parkinson's (Fernandez-Ayala et al., 2009; Humphrey et al., 2012) or Alzheimer's (El-Khoury et al., 2016) diseases.

The potential for using AOX to study mitochondrial pathophysiology at the whole-organism level in mammals has been demonstrated using lentivector transduction, creating a transgenic mouse expressing *Ciona* AOX in multiple tissues (El-Khoury et al., 2013). Notably, harmful phenotypes were again not seen, despite widespread transgene expression. However, the methodological issues arising from the nature of that model have precluded its widespread use. On insertion of AOX transgenes at multiple genomic sites in the model, none of them individually conferred expression at a high level or in all tissues. Thus, the model could not be combined with genetic disease models or other mouse mutants, could not be practically transferred into other strain backgrounds, and its long-term maintenance was essentially impossible.

Here, we report the creation of a genetically tractable transgenic mouse that ubiquitously expresses a single copy of *Ciona* AOX at substantial levels, after targeted insertion into the *Rosa26* locus. The *Rosa26* knock-in gave rise to a functional enzyme, which conferred resistance to respiratory poisons. Surprisingly, comprehensive phenotyping revealed only minor, biologically inconsequential effects of AOX expression in the *AOX^Rosa26^* mouse. The new model offers great promise as a tool for elucidating the mechanisms of mitochondrial pathology and charting the way towards future therapies.

## RESULTS

### Construction of *AOX^Rosa26^* mice

To create a genetically tractable mouse model ubiquitously expressing *Ciona* AOX, we used gene targeting into the ubiquitously active *Rosa26* locus (Hitoshi et al., 1991) in mouse embryonic stem cells (ESC) (Soriano, 1999; Srinivas et al., 2001). Previous authors have reported no detectable pathological alterations arising from insertions at this locus (Friedrich and Soriano, 1991; Zambrowicz et al., 1997), and transgene expression seems to be stable (Zambrowicz et al., 1997). To boost expression from the *Rosa26* locus, we incorporated the synthetic CAG enhancer-promoter into the construct (Fig. 1B; Fig. S1), which enhances expression several-fold (Nyabi et al., 2009; Chen et al., 2011). After verification of the insertion in ESCs by Southern blotting (Fig. S1B,C), a chimeric line was established via blastocyst injection, with subsequent elimination of the positive-selectable (neomycin resistance) cassette (Fig. S1A,B) by Flp recombination *in vivo*, following germ-line transmission. Founders were backcrossed over more than seven generations to strain C57Bl/6J, with transgene presence checked at each step by PCR (Fig. S1D). The rate of transmission of the *AOX* transgene from heterozygous parents of either sex did not significantly deviate from 50% (Fig. 1C), nor was there any significant parent-of-origin effect on litter size (Fig. 1C). The progeny sex ratio was also unaffected by the *AOX* transgene (Fig. 1D).

### AOX is ubiquitously expressed in the *AOX^Rosa26^* mouse

**Fig. 2.**
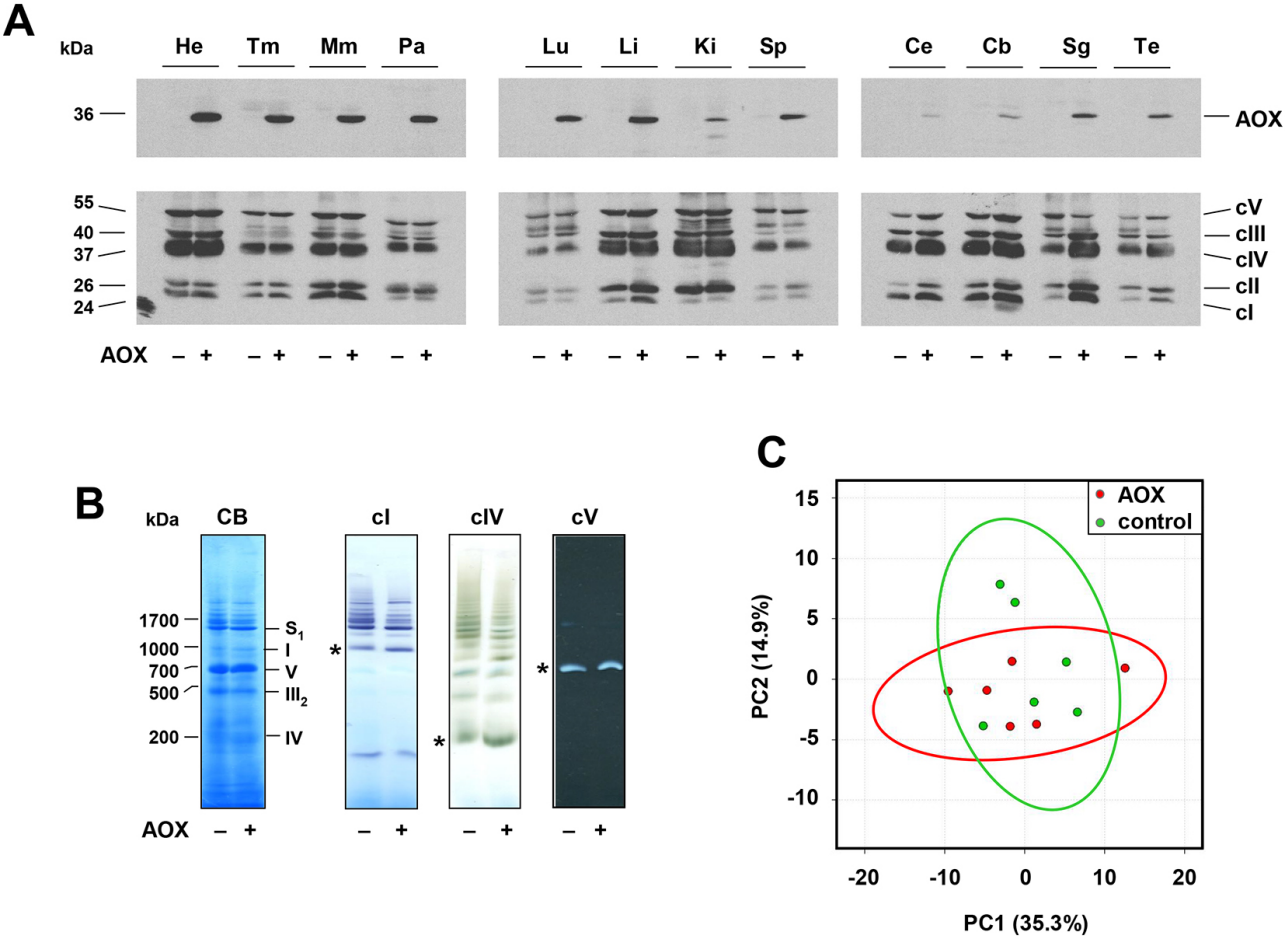
***AOX^Rosa26^* mice show broad AOX expression and normal metabolism.** (A) Western blots of 20 μg total protein extracts from the indicated tissues (He, heart; Tm, thigh muscle; Mm, masseter muscle; Pa, pancreas; Lu, lung; Li, liver; Ki, kidney; Sp, spleen; Ce, cerebrum; Cb, cerebellum; Sg, salivary gland; Te, testis) of 54-week-old male hemizygous *AOX^Rosa26^* (+) and wild-type littermate control (–) mice, probed for AOX and for representative subunits of the five OXPHOS complexes (see Materials and Methods, protein molecular weights extrapolated from markers). For Ponceau S staining of the membranes see Fig. S2B. See also Fig. S2A,C,D. (B) BNE gels of mitochondrial membrane proteins from hemizygous *AOX^Rosa26^* (+) and wild-type littermate control (–) mice, stained with Coomassie Blue (CB) or probed by in-gel histochemistry for the indicated OXPHOS complexes. * denotes the migration of the respective monomeric complexes. Assignment of mitochondrial complexes (I, cI; III_2_, dimeric cIII; IV, cIV; V, cV; S_1_, respiratory supercomplexes containing cI, dimeric cIII and one copy of cIV) is based on protein molecular weights extrapolated from the migration of the complexes from bovine heart mitochondria, whose subunit composition is known. (C) Principal component analysis of metabolome data from skeletal muscle of hemizygous *AOX^Rosa26^* (red circles) and wild-type littermate control mice (green circles). The two sets of analysed data overlap, apart from two minor outliers from the control group. See also Fig. S2H.

Northern blotting (Fig. 1E) confirmed widespread, though somewhat uneven, expression with highest *AOX* mRNA levels in heart and skeletal muscle, but lower expression in brain, taking account of the loading controls. At the protein level, expression seemed more uniform, but was again highest in heart, skeletal muscle and pancreas, and lowest in brain (Fig. 2A; Fig. S2). Brain expression was highest in newborn mice (Fig. S2C), but declined substantially by one month of age (Fig. S2C). As expected, AOX expression was higher in homozygotes compared with heterozygous animals (Fig. S2D). The enzyme was found to be associated with the membrane fraction of isolated mitochondria after carbonate extraction (Fig. S2E), albeit less tightly bound than some integral membrane proteins of the OXPHOS complexes, such as subunit 1 of cIV (Mtco1).

In each tissue tested, the expression of representative subunits of the five OXPHOS complexes was essentially unaffected by AOX expression (Fig. 2A; Fig. S2A). Moreover, the overall structure of the respiratory membrane, specifically its organization into supercomplexes, was similarly unaltered, based on blue-native electrophoresis (BNE) followed by in-gel histochemistry of heart mitochondria (Fig. 2B), and on BNE combined with western blots for OXPHOS subunits, for eight different tissues (Fig. S2F). In BNE gels, AOX itself migrated mainly at the size of a dimer and as multimers thereof (Fig. S2F,G), rather than associating specifically with any other respiratory complex. In each tissue tested, the mobility of the respiratory chain complexes detected by BNE was identical to that in controls (Fig. S2F). Principal component analysis of metabolite levels in skeletal muscle (Fig. 2C) and heart (Fig. S2H) showed no consistent effect of AOX expression, nor did any of 100 individual metabolites analyzed show any significant difference (Tables S1, S2).

### AOX is functional in *AOX^Rosa26^* mice

**Fig. 3.**
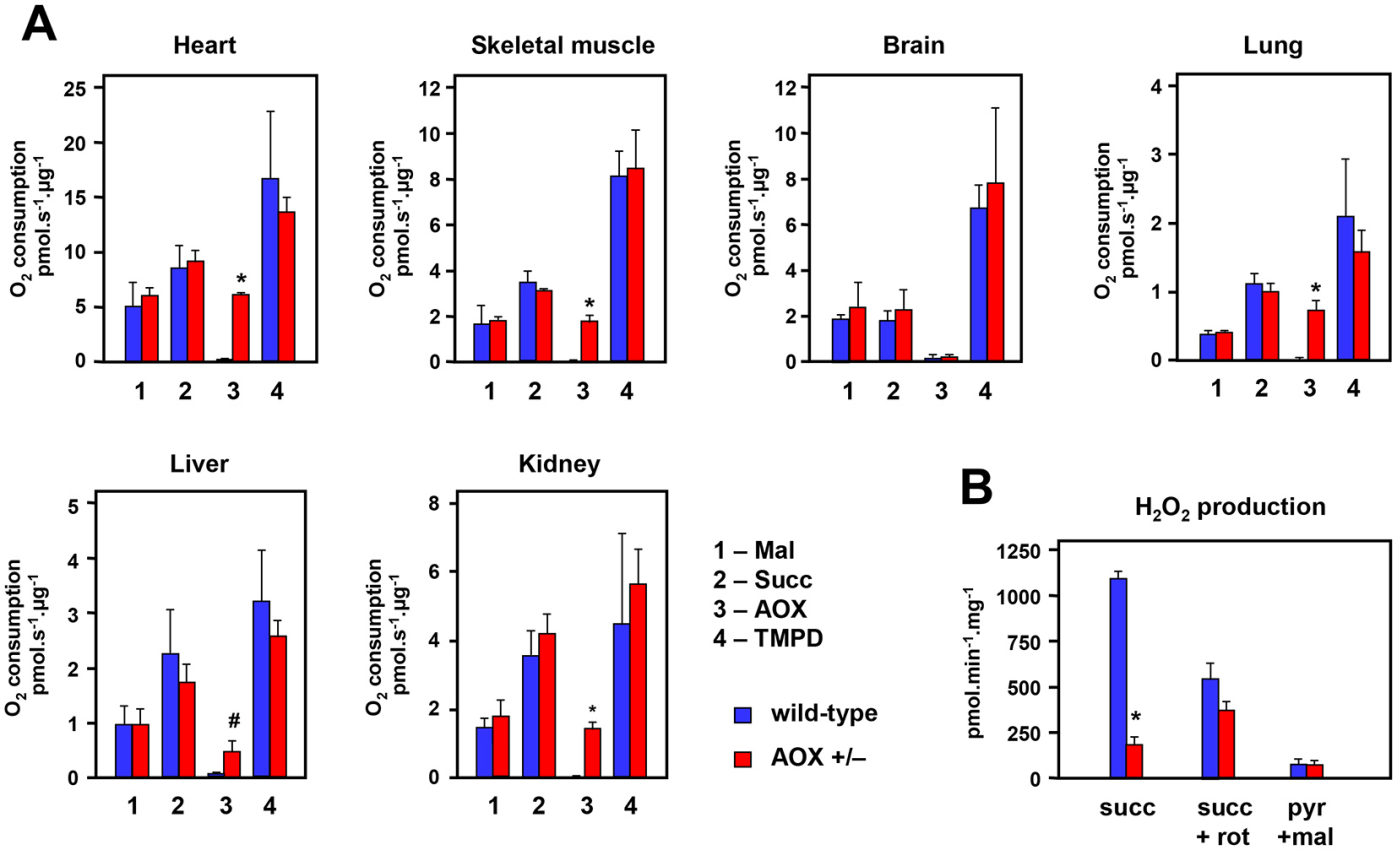
**AOX is enzymatically functional in mitochondria from *AOX^Rosa26^* mice.** (A) Respirometry (oxygen consumption in the indicated units) from isolated mitochondria prepared from the indicated tissues of hemizygous *AOX^Rosa26^* mice and wild-type littermate controls, as shown; means±s.d. of three biological replicates in each case. Note the different scales. 1 (Mal) – rotenone-sensitive oxidation of malate in the presence of glutamate, pyruvate and ADP; 2 (Succ) – antimycin A- plus n-propyl gallate-sensitive succinate oxidation; 3 (AOX) – rate of n-propyl gallate-sensitive, antimycin A-insensitive succinate oxidation; 4 (TMPD) – rate of ascorbate-reduced TMPD oxidation. For further details see Materials and Methods. See Fig. S3C for respiratory control ratio in these samples. (B) ROS production, measured as H_2_O_2_ output, from heart mitochondria of *AOX^Rosa26^* mice and wild-type littermates, as indicated, driven by the indicated substrates or inhibitors (succ, succinate; rot, rotenone; pyr, pyruvate; mal, malate). ^#^*P*<0.05 or *P<0.001 between given pairs of control and *AOX^Rosa26^* values, Student's *t-*test, means±s.d. Note that it is not possible to verify that this effect depends on the enzymatic activity of AOX, because the AOX inhibitor, n-propyl gallate, is itself a potent antioxidant.

We conducted respirometry to determine whether AOX is enzymatically functional in the *AOX^Rosa26^* knock-in mice. Mitochondria from six tissues (Fig. 3) were tested in a standard protocol for oxygen consumption in the presence of complex I-, II- and IV-linked substrates, successively using inhibitors of cI (rotenone), cIII (antimycin A), AOX (n-propyl gallate) and cIV (cyanide or azide). There were no significant differences when oxygen consumption was compared with that from mitochondria of wild-type littermates, except for substrate oxidation in the presence of antimycin A (i.e. mediated by AOX), which was significant for all tissues tested except brain, where expression was low. Mitochondria from tissues of *AOX^Rosa26^* mice other than brain showed antimycin A-resistant (AOX-dependent) oxygen consumption between 30% and 70% of the uninhibited level driven by succinate (Fig. 3A), similar also to preliminary measurements in the founder mouse (Fig. S3A). In heart mitochondria from AOX-expressing compared with control mice, antimycin A- and azide-resistant substrate oxidation was evident across a wide range of drug concentrations (Fig. S3B). Compared with littermate controls, mitochondrial ROS production driven by succinate was greatly decreased (Fig. 3B). Interestingly, this was only significant in the absence of rotenone, implicating AOX in providing an alternative pathway for succinate oxidation other than reverse electron transport through cI.

### *AOX^Rosa26^* mice exhibit normal physiology

**Fig. 4.**
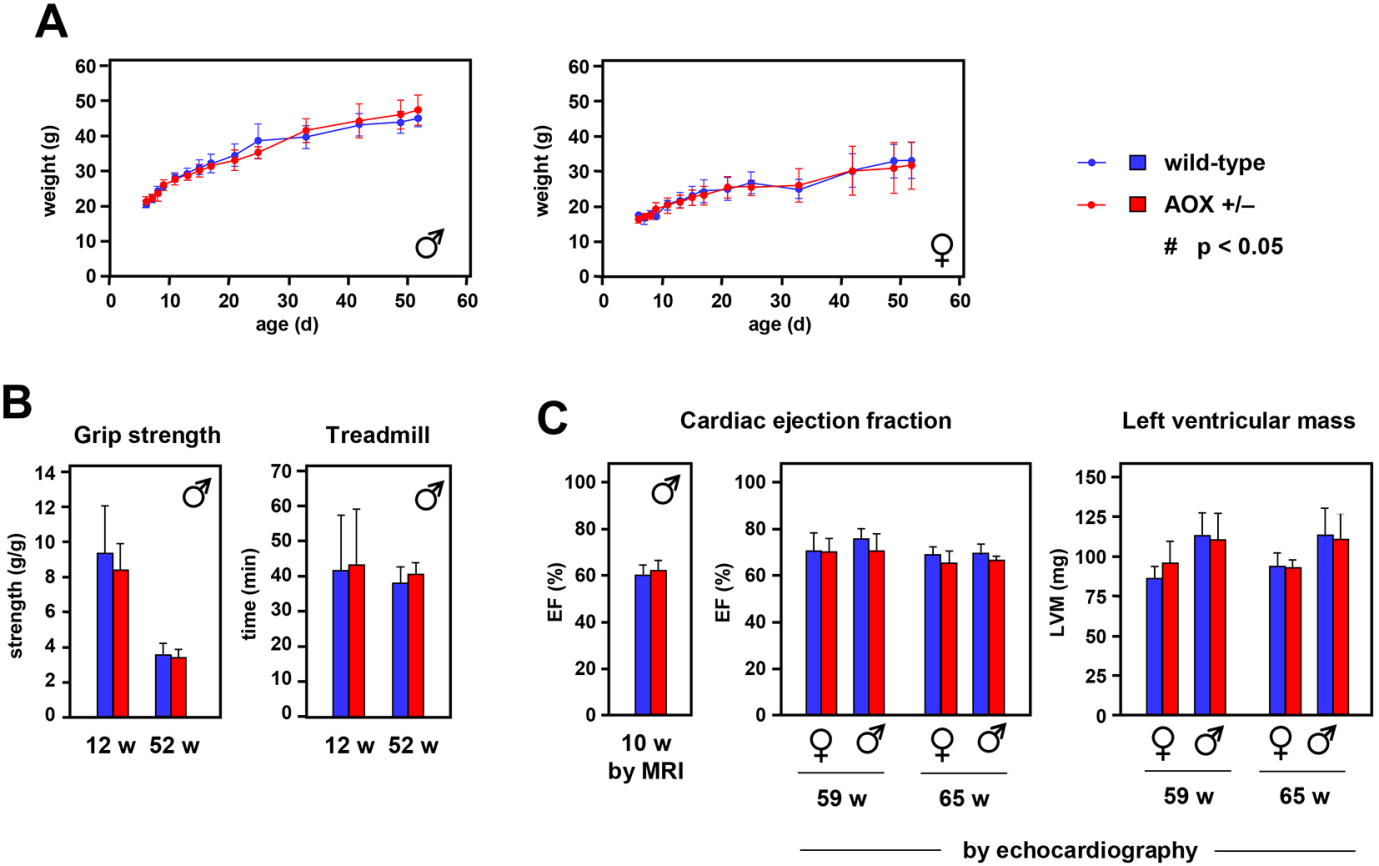
***AOX^Rosa26^* mice exhibit normal physiology.** (A) Mean weight±s.d. of hemizygous *AOX^Rosa26^* and wild-type littermate control mice of the sexes indicated, during post-natal development, *n*≥8 for each sex and genotype analysed. (B) Muscle parameters of male hemizygous *AOX^Rosa26^* and wild-type littermate control mice of the ages indicated; means±s.d. For each group analysed *n*≥4 (grip strength) or *n*≥6 (treadmill). (C) Cardiac parameters, as indicated, of hemizygous *AOX^Rosa26^* and wild-type littermate control mice of the sex and ages indicated; means±s.d., *n*≥4 for each group analysed. All data obtained by echocardiography except ejection fraction at 10 w of age, which used MRI, *n*≥5. There were no significant differences between *AOX^Rosa26^* and wild-type values for any parameter measured (Student's *t*-test, *P*>0.05).

The high level of AOX expression, capable of replacing a large fraction of electron flow when cIII/IV is inhibited, raised the question of potentially deleterious consequences under normal physiological conditions. Surprisingly, *AOX^Rosa26^* mice of both sexes were similar in size to littermate controls and gained weight normally during development (Fig. 4A). Muscle and heart functions showed no significant differences from littermate controls, based on standard assays of grip strength (Fig. 4B), treadmill performance (Fig. 4B), cardiac ejection fraction (Fig. 4C) and left ventricular mass (LVM; Fig. 4C), conducted on mice of different ages. To complement these data we implemented a comprehensive phenotyping, using the resources of the German Mouse Clinic (https://www.mouseclinic.de, search ‘phenomap’; hereafter referred to as ‘GMC Phenomap’). This analysis covered metabolic, behavioural, morphological, immunological, cardiac and neurological parameters, amongst others. None of the parameters tested showed substantial or systematic deviations from littermate controls.

### AOX confers protection against an LD50 dose of systemically delivered cyanide

**Fig. 5.**
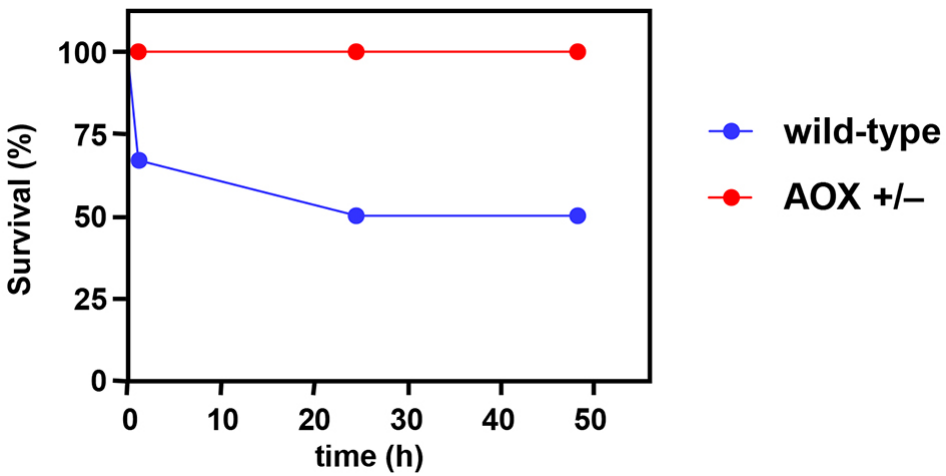
**Sampled *AOX^Rosa26^* mice are protected against cyanide toxicity *in vivo*.** Survival curves of samples of hemizygous female *AOX^Rosa26^* mice (*n*=5) and wild-type controls (*n*=6) treated systemically with KCN as described in Materials and Methods. Note that the experiment would need to be conducted on a much greater scale to generate fully reliable statistics, but is precluded by ethical considerations.

Despite the absence of any meaningful phenotype under standard (non-stressful) physiological conditions, we reasoned that the ubiquitous expression (Fig. 2) of functional AOX (Fig. 3) should confer whole-organism resistance to a respiratory poison targeting cIII or cIV. Sample cohorts of female mice were thus tested for their response to systemically administered potassium cyanide at ∼LD50 (Yamamoto, 1995), with evaluation of survival after 1, 24 and 48 h. All five *AOX^Rosa26^* transgenic mice tested survived the treatment, whereas three of six littermate controls succumbed as expected (Fig. 5). Although the sample sizes are small, hence indicative rather than definitive, the result is consistent with protection against cyanide at the whole-organism level.

## DISCUSSION

In this study we successfully engineered mice for stable, ubiquitous expression of *Ciona* AOX, via a single-copy insertion into the *Rosa26* locus, controlled by the synthetic CAG promoter. AOX protein was widely expressed and enzymatically functional when tested in the presence of antimycin A *in organello*. AOX expression produced negligible phenotypic effects under standard physiological conditions, but seemed able to protect mice from the lethal effects of injected cyanide. The *AOX^Rosa26^* mouse provides a genetically tractable tool for analyzing the pathophysiology of a wide spectrum of diseases proposed to be linked to mitochondrial respiratory dysfunction.

### The *AOX^Rosa26^* mouse is a genetically tractable model

The ‘MitAOX’ transgenic mice, previously generated by lentivector transduction (El-Khoury et al., 2013), provided a preliminary indication that widespread *Ciona* AOX expression in the mouse is not harmful. However, owing to the multi-copy nature of the inserted transgene at different genomic sites, as well as varying expression levels, MitAOX mice were not suitable for studies using genetic disease models. To avoid these problems, we created a revised model containing a single insertion of *AOX* cDNA at the *Rosa26* locus on chromosome 6. We demonstrated (Fig. 1) that the introduced AOX gene is stably transmitted in a Mendelian manner, remains active beyond at least seven generations of backcrossing to strain C57Bl/6J, shows no parent-of-origin or sex-specific lethality, and is widely expressed. The AOX transgene can, in principle, be transferred to any desired strain background suitable for combination with a given genetic model of disease, although our current analysis was confined to the C57Bl/6J genetic background. We expect that the *AOX^Rosa26^* mouse will become a versatile model for studying the nature of mitochondrial involvement in disease-like phenotypes.

### AOX seems inert under standard physiological conditions

Although AOX was enzymatically functional in the presence of antimycin A *in organello*, our data indicate that the metazoan enzyme is functionally inert under standard physiological conditions, as suggested previously (Hakkaart et al., 2006; Fernandez-Ayala et al., 2009; El-Khoury et al., 2013). Several lines of evidence support this conclusion: (1) any substantial contribution by the non-proton-motive AOX to respiratory electron flow should manifest in a significantly decreased respiratory control ratio in respirometric measurements *in organello*. However, we did not observe any significant alteration in our tissue survey at least for cI-linked substrates and within the detection limits of the method applied (Fig. S3C). (2) Inefficient mobilization of nutritional resources by AOX-expressing mice should alter metabolic parameters *in vivo*. However, there were no differences in whole body weight (Fig. 4A), fat or lean body mass determined by nuclear magnetic resonance (NMR) (see GMC Phenomap), or physiological parameters determined by indirect calorimetry, including food intake, body temperature, oxygen/CO_2_ exchange or activity (see GMC Phenomap). (3) No significant differences in heart performance were detected by electro- or echocardiography, or MRI (Fig. 4C; GMC Phenomap), although the heart is the most energy-demanding tissue and showed the highest AOX expression.

### Low AOX expression in brain

The relatively low expression of the *AOX* transgene in adult brain (Fig. 1E, Fig. 2A, Fig. S2A,D) is somewhat puzzling, given previous reports. The CAG promoter has previously been used to drive transgene expression at a high level in the mouse brain during development (Liu et al., 2014) as well as in the adult (Ida-Hosonuma et al., 2002; Kim et al., 2013), and the *Rosa26* locus efficiently drives expression in the brain (Banares et al., 2005; Hitz et al., 2007; Delaunay et al., 2009). Moreover, we also achieved substantial expression in the brains of MitAOX transgenic mice (El-Khoury et al., 2013), using the same CAG promoter.

Intriguingly, neonatal *AOX^Rosa26^* brains expressed substantially more AOX at the protein level than adults (Fig. S2C), indicating that the transgene can be active, but apparently regulated, in neural cells. At present we do not have a convincing explanation for these anomalies. However, the relatively low expression of AOX in the adult brain seemed sufficient to protect against the lethality of systemically delivered cyanide (Fig. 5), which can cross the blood-brain barrier and has major toxic effects in the central nervous system (Yamamoto and Tang, 1996; Reiter et al., 2010; Zhang et al., 2015). It will be interesting to explore in greater depth the specific physiological effects of this dose of cyanide and how these effects are modified by AOX expression.

### Lack of metabolic disturbance resulting from AOX expression

The lack of any discernible, deleterious phenotype at the whole-organism level arising from AOX expression (Fig. 4; supplementary Data), mirrors the lack of biochemical disturbance in the *AOX^Rosa26^* mouse. This was the case even in heart (Fig. 2A,B) and skeletal muscle (Fig. 2A) tissues showing high levels of AOX expression. The highly proteinaceous inner mitochondrial membrane is organized into different subcompartments with distinct structures, protein composition and biochemical functions (Vogel et al., 2006). In particular, the supramolecular organization of the OXPHOS system in supercomplexes is generally considered to maximize the efficiency of electron flow (Acín-Pérez et al., 2008; Chaban et al., 2014). We observed no structural (Fig. 2B) or functional (Fig. 3A) disturbance of the endogenous respiratory membrane upon AOX expression, which might reflect natural properties of *Ciona* AOX, enabling it to reside in the mitochondria of its parent species. Transgenic AOX seems to form homomeric complexes, rather than associating with (and potentially disrupting) other OXPHOS complexes. The electrophoretic mobility of the standard OXPHOS complexes was indistinguishable from that in controls, in all tissues tested (Fig. 2B; Fig. S2F,G). Our findings imply that these multimers are themselves benign, although it remains unknown whether AOX is structurally arrayed in a similar manner in its natural context in *Ciona*. We reason that, by remaining uncomplexed with other respiratory chain components, the enzyme would be functionally adapted to act as a sink for electrons transferred from diffusible quinols in the inner mitochondrial membrane. Quinone reduction might arise from the operation of diverse dehydrogenases, including, for example, cII, electron transferring flavoprotein dehydrogenase, the mitochondrial isoform of glycerol-3-phosphate dehydrogenase and dihydroorotate dehydrogenase. Under normal physiological conditions, diffusible quinols would be efficiently mopped up by (dimeric) cIII, whether alone or attached to cIV as a supercomplex. As in plants (Hoefnagel and Wiskich, 1998; Castro-Guerrero et al., 2004), AOX would only become active at high quinol concentrations, reflecting its lower affinity for quinols than cIII. Thus, AOX would be brought into play only when quinols accumulate as a result of inhibition or overload of the standard respiratory pathway, as inferred previously in human cells (Dassa et al., 2009). This hypothesis is also consistent with our observation that AOX expression drastically decreases mitochondrial ROS production by heart mitochondria in the presence of high levels of succinate (Fig. 3B), which promotes reverse electron flow through cI (Chouchani et al., 2014). Our observations suggest that AOX might have dramatic consequences under stress, especially in heart and other tissues where it is highly expressed. Tests of this hypothesis will also reveal whether AOX could have beneficial roles in future therapies (El-Khoury et al., 2014). A first demonstration of the utility of *AOX^Rosa26^* mice has recently been published (Mills et al., 2016), in which AOX was shown to confer resistance against lethality in models of bacterial sepsis. Further trials, combining *AOX^Rosa26^* with specific genetic disease models, should reveal the extent to which AOX can alleviate the pathophysiology of respiratory chain dysfunction. The *AOX^Rosa26^* mouse model should have wide applications and is available for the research community, upon request.

## MATERIALS AND METHODS

### Construction of targeting vector

Standard cloning and recombineering procedures (Liu et al., 2003; Warming et al., 2005) were used to assemble the pRosa26-Aox targeting vector. Briefly, PL451 was adapted to serve as AOX entry vector, by integrating homology arms for the mouse *Rosa26* locus (PL451-Rosa26) upstream and downstream of the neomycin selection cassette. *Ciona* AOX (Hakkaart et al., 2006) was integrated upstream of the selection cassette, and used to co-transform recombineering-competent *Escherichia* *coli* (EL250) with a *Rosa26*-targeting plasmid (pRosa26-DTA). Positive clones were selected by kanamycin resistance (pRosa26-Aox), verified by PCR and sequencing, and electroporated into v6.5 ESCs following linearization with *Sal*I. After negative (DTA, Diptheria toxin A) and positive (G418) selection, homologous integration was verified by Southern blotting (Koetsier et al., 1993) using gene-specific restriction enzymes and probes to distinguish the wild-type and manipulated alleles (see supplementary Materials and Methods and Fig. S1B,C for further details).

### Creation of transgenic mice

ESC clones positive for integration were injected into blastocysts and transferred to pseudopregnant mice. Chimeric males were then backcrossed onto the C57Bl/6J strain background to generate heterozygous animals, and subsequently bred with mice ubiquitously expressing FLP recombinase (Rodríguez et al., 2000), in order to delete the neomycin selection cassette. Mice were backcrossed (>7 generations) to C57Bl/6J females to obtain a clean genetic background for all subsequent studies.

### PCR genotyping of *AOX^Rosa26^* mice

Crude DNA for genotyping was extracted from ear punches or tail cuts by standard methods (proteinase K treatment, isopropanol precipitation and overnight resuspension in TE at 56°C). Multiplex PCR genotyping was carried out using primers Aox 317 s: 5′-GCGATGCAAGATGGAGGGTA-3′ plus Aox 317 as: 5′-TGAATCCAACCGTGGTCTCG-3′ for *AOX*, and Rosa26_wt s: 5′-GACCTCCATCGCGCACTCCG-3′ plus Rosa26_wt as: 5′-CTCCGAGGCGGATCACAAGC-3′ for the wild-type *Rosa26* locus, giving respective products of 317 and 523 bp. PCR reactions of 20 μl contained 4 pmol of each primer, DMSO at 2% and 0.2 μl DyNazyme II (Thermo Fisher Scientific), with cycle parameters of initial denaturation at 95°C for 5 min, then 39 cycles of denaturation at 95°C for 20 s, annealing at 56°C for 30 s and extension at 72°C for 60 s, with final extension step at 72°C for 10 min, followed by 1.5% agarose gel electrophoresis. See Fig. S1D for example gel.

### RNA analysis

RNA was prepared from dissected mouse tissues by bead homogenization in 700 μl (>10 volumes) of Trizol reagent (Sigma). After incubation for 5 min at room temperature, samples were gently extracted with 0.2 volumes of chloroform and centrifuged at 12,000 ***g****_max_* for 15 min at 4°C. The upper (aqueous) phase was decanted and RNA recovered by isopropanol precipitation and centrifugation. Using standard procedures (Sambrook et al., 1989), air-dried RNA pellets were resuspended in 20 μl RNase-free water, fractionated on formaldehyde-agarose gels, blotted to Hybond-N+ membrane (GE Healthcare) in 10×SSC and hybridized to end-labelled DNA oligonucleotide probes for *AOX*, mitochondrial 12S and cytosolic 18S rRNA, and *Atp5b* mRNA, respectively 5′-CTTGACCCACTGTTTCTCATCTAGCCG-3′, 5′-CATGGGCTACACCTTGACCT-3′, 5′-TCGAACCCTGATTCCCCGTCACCC-3′ and 5′-GGTGAATATGACCATCTCCCAGAACAAGC-3′.

### Protein analysis

For protein extraction, small pieces of fresh or frozen tissue from dissected organs were placed in 500 μl of lysis buffer (50 mM Tris/HCl, 150 mM NaCl, 1 mM EDTA, 1% Triton X-100, pH 7.4), containing a dissolved protease inhibitor cocktail tablet (Pierce), in a 5 ml tube on ice. After homogenization using a POLYTRON PT 1200 E Manual Disperser (Ecoline), samples were incubated on ice for 30 min followed by centrifugation at 14,000 ***g***_*max*_ for 5 min at 4°C. Supernatants were saved and protein concentration was measured using Bradford reagent (Bio-Rad) before dilution into SDS-PAGE sample buffer for electrophoresis on SDS 12% polyacrylamide gels. After semi-dry transfer to PROTRAN nitrocellulose membranes (PerkinElmer), western blots were probed using primary antibodies for AOX [customized rabbit antibody, 21st Century Biochemicals (Fernandez-Ayala et al., 2009), 1:40,000 in Tris-buffered saline (TBST) containing 5% BSA] with secondary antibody peroxidase-conjugated AffiniPure goat anti-rabbit IgG (Jackson ImmunoResearch, 111-035-144, 1:20,000). After stripping by two 20 min washes with 100 mM β-mercaptoethanol, 2% SDS, 62.5 mM Tris-HCl (pH 6.7), each followed by blocking with TBST containing 5% milk for 30 min, blots were reprobed for representative subunits of the OXPHOS complexes, using Total OXPHOS Cocktail antibody [Abcam, ab110413, 1:250; visualizing Sdhb (cII), Uqcrc2 (cIII), Mtco1 (cIV) and Atp5a (cV)], plus an antibody against complex 1 subunit Ndufs3 (Mitosciences, ab14711, 1:4000), both detected with peroxidase-conjugated AffiniPure goat anti-mouse IgG (Jackson ImmunoResearch, 115-035-146, 1:1000) as secondary antibody. Chemiluminescent detection used 20× LumiGLO Reagent and 20× Peroxide from Cell Signaling Technology, according to manufacturer's recommendations. Enrichment of mitochondrial membranes, solubilisation of mitochondrial complexes and BNE were carried out as described (Wittig et al., 2006; Heidler et al., 2013). Mitochondrial complexes were stained with Coomassie Blue (Wittig et al., 2006) and specific in-gel histochemical staining for cI, cIV, and cV was performed as described previously (Wittig et al., 2007). For immunodetection, BNE-gels were blotted onto PVDF membranes and probed with antibodies against AOX (1:50,000) or mitochondrial complexes (MitoProfile Total OXPHOS Rodent WB Antibody Cocktail, Mitosciences, ab110413, 1:250) and cIV (1:1000; Heidler et al., 2013).

### Metabolomics

Metabolite analysis was conducted as described previously (Nikkanen et al., 2016), using skeletal muscle from six hemizygous *AOX^Rosa26^* and six wild-type littermate control mice (8-week-old males, all culled at a single time in the morning). Briefly, targeted metabolomics was implemented by ultra-performance liquid chromatography tandem mass-spectrometry using a Waters XEVO-TQ-S mass spectrometer. Metabolites extracted with acetonitrile were separated by hydrophilic liquid-interaction chromatography, then analysed spectrometrically by multiple reaction monitoring. Raw data were collected and analysed with TargetLynx software (Waters), and metabolites quantified using internal standards and calibration curves. For full details, see supplementary Materials and Methods.

### Bioenergetic experiments

For respirometry of mitochondria from different tissues, mice were euthanised by cervical dislocation and organs were dissected and collected into ice-cold PBS. Soft tissues were fine chopped (1 mm^3^) in ice-cold PBS and hand-homogenized in 3 ml re-suspension buffer [225 mM sucrose, 75 mM D-mannitol, 10 mM Tris/HCl, 1 mM EGTA, 1 mg/ml bovine serum albumin (BSA), pH 7.4], using a glass-teflon homogenizer (tight-fitting pestle). Hard tissues (heart, skeletal muscle and kidney), chopped to a similar size, were pre-treated with 3 ml (∼10 volumes) ice-cold trypsin-EDTA [500 μg/ml trypsin (Difco), 0.5 mM EDTA, 10 μg/ml phenol red, pH 7.4] for 10 min, followed by blocking with 300 μl foetal bovine serum (Gibco/Life Technologies) and recovery by low-speed centrifugation (40 ***g****_max_*, 1 min, 4°C) before homogenization. Homogenates were centrifuged at 1300 ***g****_max_* for 5 min at 4°C, after which supernatants were collected and re-centrifuged at 17,000 ***g****_max_* for 15 min at 4°C. The mitochondrial pellet was resuspended, according to its size, in 75-250 μl ice-cold MiR05 buffer [0.5 mM EGTA, 3 mM MgCl_2_, 60 mM lactobionic acid (Aldrich, buffered to pH 7.0 with 5 M KOH), 20 mM taurine (Sigma), 10 mM KH_2_PO_4_, 20 mM HEPES/KOH, 110 mM sucrose and 1 g/l fatty-acid free BSA (Sigma), pH 7.2 at room temperature] and stored on ice until respirometry. Mitochondrial protein content was assayed using Bradford reagent (Bio-Rad). Respirometry, using an O2K oxygraph (Oroboros), was conducted in MiR05 buffer in a 2 ml chamber, to which was added 50 or 100 µg of mitochondria according to the tissue. Substrates and inhibitors were added in the following order: (1) 5 mM sodium pyruvate+5 mM sodium glutamate+5 mM sodium malate, (2) 4 mM ADP, (3) 150 nM rotenone (Sigma), (4) 17 mM sodium succinate, (5) 22.5 ng/ml antimycin A (Sigma), (6) 200 µM n-propyl gallate (nPG, Sigma), (7) 0.5 mM N,N,N′,N′-tetramethyl-p-phenylenediamine (TMPD, Sigma)+2 mM sodium L-ascorbate, (8) 100 mM NaN_3_ or 1 mM KCN. The flux values [pmol/(s×ml)] obtained from the trace were normalized to the amount of mitochondrial protein. For measurements of ROS production, mouse heart mitochondria were isolated essentially as described (Mela and Seitz, 1979), with minor modifications: tissue was minced in 225 mM mannitol, 20 mM MOPS, 75 mM sucrose, 1 mM EGTA, 0.5 mM dithiothretol, pH 7.4 and hand-homogenized in 10 ml/g tissue of the same buffer containing 0.05% Nagarse (Sigma). After addition of 30 ml of the original buffer, the homogenate was centrifuged at 2000 ***g****_max_* for 4 min at 4°C. The supernatant was passed through cheesecloth and re-centrifuged at 12,000 ***g****_max_* for 10 min. The resulting pellet was resuspended in 225 mM mannitol, 20 mM MOPS, 75 mM sucrose, 0.1 mM EGTA, 75 mM KCl, pH 7.4. Mitochondrial protein content was determined using the bicinchoninic acid assay (Wiechelman et al., 1988), with BSA as standard. ROS production under conditions used for respirometry was measured fluorimetrically using 5 µM Amplex Red (Hydrogen Peroxide Assay Kit, Thermo Fisher Scientific) and 3 units/ml horseradish peroxidase at 30°C, using a Carry Eclipse fluorimeter (Varian) with excitation at 560 nm and detection at 590 nm (Zhou et al., 1997).

### Mouse phenotyping

Mouse body weight was measured using a small electronic balance suitable for rodents. Grip strength was measured using the BIO-GS3 apparatus (Bioseb). Mice were placed on the platform until all four limbs were engaged on the grid, and then pulled to measure the force generated. The mean of three measurements was normalised to body weight (g/g) for each animal tested. All animals were trained for three successive days before the actual experiment. Endurance running was measured as previously (Yatsuga and Suomalainen, 2012), as the run time on a standard running belt (Exer-6M Treadmill, Columbus Instruments), set to reach a speed of 6.5 m/min in steps of 0.5 m/min every 3 min. A stay of more than 5 s on the electrified motivation grid (0.5 mA current) was considered as the end point of each test. Cardiac parameters (ejection fraction, left ventricular mass) were determined by echocardiography (Vevo 2100 system, FujiFilm VisualSonics Inc.) or, where indicated in figure legends, by magnetic resonance imaging (MRI), performed essentially as described elsewhere (Ziebart et al., 2008). MRI data were analysed using OsiriX Imaging Software (http://www.osirix-viewer.com/index.html). Comprehensive phenotyping by the German Mouse Clinic (GMC) was conducted using the protocols described and referenced at https://www.mouseclinic.de (search ‘phenomap’). In all tests, mouse genotypes were blinded to the experimenter and verified subsequently.

### Systemic administration of cyanide

The procedure was implemented under contract by Luria Scientific Industries, Herzliya, Israel (responsible scientist Dr Iris Maimon). Mice, whose genotypes were blinded to the experimenter, were anesthetized with 3% isoflurane in an induction chamber, after which anaesthesia was maintained by 2% isoflurane using a nose cone. Core temperature was kept at 36.5°C using a heating pad. KCN was dissolved in distilled water at 10 mg/ml and delivered by IP injection to the mice at 8.5 mg/kg. Animals were observed for 48 h for the onset of death, defined as apnea without further respiratory effort or movement or palpable cardiac pulsation.

### Ethical permits

All mouse breeding and experiments were approved by the national ethical committee in Finland, under permits ESAVI/8766/04.10.07/2015 and ESAVI/2954/04.10.07/2015. Mouse experiments conducted under contract by Luria Scientific Industries were approved by IACUC under assurance 7433J45, 07/22/2015. Maintenance of mice in Magdeburg was in accord with procedures specified by the Animal Health and Care Committees of the Otto-von-Guericke University, Magdeburg, and of the State of Sachsen-Anhalt, Germany.

### Image processing

Images were optimized for brightness and contrast and cropped for clarity. No other manipulations such as gamma corrections were made, nor was any relevant information excluded by cropping. Full, original gel images are available on request.
